# Predicting necrosis in residual mass analysis after retroperitoneal lymph node dissection: a retrospective study

**DOI:** 10.1186/1477-7819-10-203

**Published:** 2012-09-28

**Authors:** Eduardo de Paula Miranda, Daniel Kanda Abe, Adriano João Nesrallah, Sabrina Thalita dos Reis, Alexandre Crippa, Miguel Srougi, Marcos Francisco Dall’Oglio

**Affiliations:** 1 Division of Urology, University of Sao Paulo Medical School and Cancer Institute of Sao Paulo, ICESP, São Paulo, SP, Brazil; 2 Division of Urology, University of São Paulo Medical School, São Paulo, Brazil

**Keywords:** Testicular cancer, Retroperitoneal lymph node dissection, Necrosis, Teratoma

## Abstract

**Background:**

Recent studies have demonstrated that pathological analysis of retroperitoneal residual masses of patients with testicular germ cell tumors revealed findings of necrotic debris or fibrosis in up to 50% of patients. We aimed at pursuing a clinical and pathological review of patients undergoing post chemotherapy retroperitoneal lymph node dissection (PC-RPLND) in order to identify variables that may help predict necrosis in the retroperitoneum.

**Methods:**

We performed a retrospective analysis of all patients who underwent PC-RPLND at the University Hospital of the University of São Paulo and Cancer Institute of Sao Paulo between January 2005 and September 2011. Clinical and pathological data were obtained and consisted basically of: measures of retroperitoneal masses, histology of the orchiectomy specimen, serum tumor marker and retroperitoneal nodal size before and after chemotherapy.

**Results:**

We gathered a total of 32 patients with a mean age of 29.7; pathological analysis in our series demonstrated that 15 (47%) had necrosis in residual retroperitoneal masses, 15 had teratoma (47%) and 2 (6.4%) had viable germ cell tumors (GCT). The mean size of the retroperitoneal mass was 4.94 cm in our sample, without a difference between the groups (*P* = 0.176). From all studied variables, relative changes in retroperitoneal lymph node size (*P* = 0.04), the absence of teratoma in the orchiectomy specimen (*P* = 0.03) and the presence of choriocarcinoma in the testicular analysis after orchiectomy (*P* = 0.03) were statistically significant predictors of the presence of necrosis. A reduction level of 35% was therefore suggested to be the best cutoff for predicting the absence of tumor in the retroperitoneum with a sensitivity of 73.3% and specificity of 82.4%.

**Conclusions:**

Even though retroperitoneal lymph node dissection remains the gold standard for patients with residual masses, those without teratoma in the primary tumor and a shrinkage of 35% or more in retroperitoneal mass have a considerably smaller chance of having viable GCT or teratoma in the retroperitoneum and a surveillance program could be considered.

## Background

Testicular cancer has become one of the most curable solid neoplasms and serves as a paradigm for the multimodal treatment of malignancies. The appropriate integration of chemotherapy, retroperitoneal lymph node dissection (RPLND) and observation for the management of testis cancer has resulted in overall survival rates greater than 90% [1,2].

RPLND plays an important role in the management of patients with testicular germ cell tumors (GCTs), especially in those with residual masses after chemotherapy [3]. To date, series have demonstrated that pathological analysis of these masses reveals findings of necrotic debris or fibrosis in 40% to 50% of patients, teratoma in 35% to 40% and viable malignant cells in 10% to 15% of patients [4].

Even though early recognition and resection of teratoma in the retroperitoneum after chemotherapy have been accompanied by excellent prognosis, once it was initially thought to represent a benign course when present in the retroperitoneal space, but the untreated disease may have a lethal potential due to progressive local growth or malignant transformation, not to mention its classical unresponsiveness to conventional cisplatin-based chemotherapy regimens [3-6]. As for residual viable GCT, the consequence of its incomplete resection is certain disease progression [7]. Therefore, a more aggressive approach should always be considered when treating patients with teratoma or viable GCT in the retroperitoneum [7,8].

Thus, the appropriate approach to residual masses following chemotherapy remains a controversial issue, since the literature has shown that as many as half of resected masses are basically composed of necrosis or fibrotic tissue and any sort of adjuvant therapy could be waived [9]. In order to avoid a great number of apparently unnecessary post-chemotherapy RPLND (PC-RPLND), many studies have tried to develop algorithms to predict the presence of necrosis in the retroperitoneum. However, currently predictive models and imaging modalities cannot reliably predict the pathological finding of necrosis/fibrosis at PC-RPLND [9].

Some authors have established that patients without teratoma in the primary tumor and a shrinkage of 90% or more in retroperitoneal mass had little chance of having viable GCT or teratoma and could be safely put under a surveillance program with periodic imaging scans [10]. However, prospective analyses have demonstrated that approximately 30% of patients will harbor teratoma or viable malignancy even with normal post-chemotherapy computed tomography (CT) results and no teratoma in the primary tumor [9].

The purpose of this work is to pursue a clinical and pathological review of patients undergoing PC-RPLND at a reference university oncology center in Brazil, in order to identify variables that may help predict the histological finding of necrosis in the retroperitoneum and perhaps establish a differentiated surveillance protocol.

## Methods

We performed a retrospective analysis of all patients from our computerized database who underwent PC-RPLND at our service between January 2005 and May 2011. Patients were operated on after having undergone three to four cycles of primary chemotherapy with bleomycin, etoposide and cisplatin.

Clinical and pathological data were obtained and consisted basically of measures of retroperitoneal masses, serum tumor markers, histology of the orchiectomy specimen, tumor marker values and retroperitoneal nodal size before and after chemotherapy.

The presence of either immature or mature teratoma in the resected specimen, as well as teratoma with malignant transformation, was considered part of the same group. Choriocarcinoma, yolk sac tumors, embryonal carcinoma and seminoma were considered viable GCTs. All histological findings were submitted to quantitative and qualitative analysis.

Post-chemotherapy alpha-fetoprotein (AFP) and lactate dehydrogenase (LDH) levels were registered as a continuous variable while human chorionic gonadotropin (hCG) levels were considered a categorical variable ranging from undetectable to detectable when serum concentrations were greater than 3 mIU.

Retroperitoneal nodal size before and after chemotherapy was determined by the longest transverse diameter of the largest mass on CT imaging. Relative change in nodal size before and after chemotherapy was calculated by dividing post-chemotherapy nodal size by pre-chemotherapy nodal size and was analyzed as a continuous variable.

Multiple variables were analyzed independently in order to establish any predictive value for finding necrotic tissue in the retroperitoneum. Statistical analysis was performed using the Statistical Package for Social Sciences (SPSS, version 12.0, Chicago, IL, USA), applying the Mann–Whitney *U* test for non-parametric variables and the Fisher's exact test for categorical variables, with the level of significance set at *P* < 0.05.

A cut-off level for predicting necrosis at retroperitoneal residual mass analysis was sought by constructing a receiver operating characteristic (ROC) curve of all significant variables, which were generated using graphical visualization in our statistical software. This study was carried-out in accordance with the Ethics Committee regulations.

## Results and discussion

We gathered information on a total of 32 patients, who were 18- to 49-years old (mean age 30.5) and harbored seminomatous, nonseminomatous or mixed tumors in the testis tissue in different clinical stages. Three patients (9.4%) had seminoma, five (16%) had pure nonseminoma (choriocarcinoma, yolk sac tumors, embryonal carcinoma, teratoma, teratocarcinoma) and 24 (75%) harbored mixed nonseminomatous GCT (NSGCT). None of the patients with seminoma had normal AFP levels, indicating that these patients may have had tumors with similar biological behavior to those with NSGCT.

At diagnosis, seven (22%) patients were classified as having a stage I disease, while 21 (66%) were stage II and four (12.5%) were stage III. Clinical stage I patients were individuals with longer follow-up, who presented with retroperitoneal disease nonresponsive to chemotherapy and ultimately underwent PC-RPLND.

Pathological analysis in our series demonstrated that 15 (47%) patients had necrosis in residual retroperitoneal masses, 15 had teratoma (47%) and two (6%) had viable GCT: one seminoma and one yolk sac tumor. For statistical reasons and in alignment with the aims of this study, we divided those patients into two groups, assembling patients with viable GCT and teratoma and analyzing them as one group. Mean size of the retroperitoneal mass was 4.94 cm in our sample, 3.79 cm in the group of necrosis and 5.96 cm in the group of teratoma and viable GCT (*P* = 0.176). There was also no difference between groups regarding stratification of nodal size, as shown in Table 1.

**Table 1:** Distribution according to nodal size in men undergoing PC-RPLND

	**Necrosis**	**Teratoma or Viable GCT**	**Total**	***P* Value**
Number of patients (%)	15 (47)	17 (53)	32	
Mean size (cm)	3.79	5.96	4.94	0.176
Node size number (%)				
1 cm or less	1 (7)	1 (6)	2 (6)	0.514
1 to 2 cm	3 (20.0)	0 (0.0)	3 (10)	0.091
2to 5 cm	8 (53)	9 (53)	17 (53)	0.630
Greater than 5 cm	3 (20.0)	7 (41)	10 (31)	0.182

Data are shown as number (%). GCT, germ cell tumor; PC-RPLND, post-chemotherapy retroperitoneal lymph node dissection.

Primary tumor histology revealed embryonal cell carcinoma in 56%, seminomatous elements in 16%, yolk sac tumor in 19% and teratomatous elements in 56% of the patients. Of the 18 patients with teratomatous elements in the primary tumor, 13 (72%) had teratoma in the retroperitoneum at PC-RPLND. Even in the absence of teratoma in the primary tumor, teratoma was present in the retroperitoneum in five patients (16%).

When comparing pathological analysis of primary tumor specimens, we found a statistical difference when comparing the prevalence of teratoma and choriocarcinoma. Other findings, such as the presence of seminoma, yolk sac tumor, embryonal carcinoma, endodermal sinus tumor lymph vascular invasion (LVI), rete testis invasion and spermatic invasion were similar between groups.

Univariate analysis of quantitative components at orchiectomy histology, retroperitoneal node size with its relative measures and serum markers are shown in Table 2.

**Table 2:** Univariate analysis predicting necrosis at PC-RPLND

	**Necrosis**	**Teratoma or viable GCT**	***P* Value**
Components at orchiectomy histology (%):			
Embryonal	22.7 ± 30.1	32.9 ± 29.7	0.370
Yolk sac	4.3 ± 11.5	4.1 ± 8.0	0.737
Teratoma	12.7 ± 23.7	13.8 ± 17.1	0.502
Endodermal sinus	1.7 ± 4.5	5.0 ± 11.7	0.628
Retroperitonial node size			
RP node cm before chemotherapy	6.5 ± 4.1	6.1 ± 5.5	0.849
RP node cm after chemotherapy	3.2 ± 1.8	5.3 ± 4.8	0.331
Relative change in RP node size	31.9 ± 48.3	9.4 ± 38.8	0.044
Absolute change in RP node size	3.2 ± 2.9	0.78 ± 2.9	0.053
Relative reduction in RP node	56.4 ± 14.0	26.2 ± 20.1	<0.001
Relative enlargement in RP node	35.6 ± 44.1	60.1 ± 22.5	0.425
Serum markers			
hCG after chemotherapy	0.0	0.0	**
AFP after chemotherapy	1.3 ± 0.4	22.1 ± 49.3	<0.001
Relative change in AFP after chemotherapy	15.7 ± 46.7	−83.7 ± 5.13	0.075
LDH after chemotherapy	307.7 ± 60.7	324.0 ± 65.8	0.399
Relative change in LDH after chemotherapy	1.5 ± 15.9	0.68 ± 27.4	0.915

** unable to calculate. Data expressed as mean ± standard deviation. AFP, alpha-fetoprotein; GCT, germ cell tumor; hCG, human chorionic gonadotropin; LDH, lactate dehydrogenase; PC-RPLND, post-chemotherapy retroperitoneal lymph node dissection; RP, retroperitoneal.

Even though the presence of teratoma in the primary tumor was an important negative predictor for the finding of necrosis, the quantitative analysis has proven to be statistically irrelevant.

While relative reduction in mass size after chemotherapy has been shown to be an important predictor of necrosis, even when considering patients not responding to chemotherapy, absolute reduction and enlargement of the residual mass did not show a significant difference between groups.

There was a statistical difference in AFP levels between groups; however, when comparing relative changes in AFP levels after chemotherapy, no difference was found. Comparison of LDH levels and their relative changes was nonsignificant. We were unable to compare hCG levels between groups because we only had two patients whose hCG levels were not undetectable.

Of all the studied variables, relative changes in retroperitoneal lymph node size (*P* =0.04), the absence of teratoma in the orchiectomy specimen (*P* =0.03) and the presence of choriocarcinoma in the testicular analysis after orchiectomy (*P* =0.03) were statistically significant predictors of the presence of necrosis in the retroperitoneum.

ROC curves were built for variables that were independent predictors of necrosis at PC-RPLND. Even though the size of the retroperitoneal mass after chemotherapy showed no statistical difference, we also built a curve for it. The variable relative reduction after chemotherapy was the only one with predictive value according to the Youlden index. An area under the curve (AUC) of 0.710 was obtained and a reduction level of 35% was therefore suggested to be the best cutoff for predicting the absence of tumor in the retroperitoneum with a sensitivity of 73% and a specificity of 82%. ROC curves of isolated size of retroperitoneal mass, absence of teratoma and presence of choriocarcinoma did not indicate significant findings.

In our institution, relative change in retroperitoneal node size, absence of teratoma in the orchiectomy specimen and the presence of choriocarcinoma in the testicular analysis after orchiectomy were statistically significant predictors of the presence of necrosis in the retroperitoneum and a surveillance program could be considered, given the uncertainty in predicting the histology of residual masses after chemotherapy in patients with metastatic testicular tumors.

Analysis of residual retroperitoneal masses after chemotherapy is being increasingly regarded as a fundamental issue, not only for orienting adjuvant therapies, but also because it has prognostic implications [11]. Outcome assessments in patients with NSGCT have demonstrated that incomplete resection of residual retroperitoneal masses, the size of residual retroperitoneal masses and the finding of teratoma and viable GCT at RPLND independently predict disease progression and relapse [11,12].

The size of residual retroperitoneal masses after chemotherapy has traditionally been considered when choosing the subsequent treatment modality [9]. Studies have shown that residual masses smaller than 2 cm are considered one of the most significant predictors for finding necrosis at PC-RPLND at logistic regression [4]. Furthermore, a number of investigators continue to base the decision to perform PC-RPLND on residual mass size alone, obviating PC-RPLND in patients with residual masses of 1 cm or less [13].

However, recent studies have shown that after chemotherapy a third of retroperitoneal masses of <2 cm harbored either teratoma or viable GCT [14]. Even though we found a trend of having only necrosis in the retroperitoneum for masses <2 cm (*P* = 0.09), in the present study residual mass size alone has not appeared to be a good predictor of necrosis, with nonsignificant size differences between groups and an underrated AUC in ROC curves. In addition, one case of teratoma in a 1 cm mass was registered in our sample. Therefore, we advocate that the decision of not operating on patients cannot be based on mass size alone due to the lack of consensus in the literature [9,10,13,14].

Traditional series report necrotic debris or fibrosis in 40% to 50%, teratoma in 35% to 40% and viable malignant cells in 10% to 15% of patients [3]. In more recent analyses, the incidence of residual microscopic teratoma in the retroperitoneum has decreased to approximately 20% to 25%, with an increase of necrotic tissue findings of up to 60% and stable rates of viable GCT [15]. It has been reported that an increase in the proportion of necrosis is generally attributed to stage migration and the use of more effective chemotherapy regimens, especially in patients achieving a complete response to chemotherapy [15].

In our series we found a distribution pattern similar to former studies, with almost 50% of patients harboring teratoma in the retroperitoneal histology at RPLND, which may suggest that our chemotherapy regimens have been having inferior rates of complete responders or simply because our group is composed of patients in more advanced stages.

Teratoma-negative primary tumor and volumetric regression of at least 90% after chemotherapy have been described as being highly predictive of harboring necrosis only at PC-RPLND [9,16]. Other series include normal serum tumor markers, and node size < 2 cm, the presence of yolk sac tumor or embryonal carcinoma on primary tumor and lymph vascular invasion, among others [9,10,15-17]. On the other hand, some authors have been unable to identify variables to be highly predictive of harboring necrosis only at PC-RPLND [15,18]. Nevertheless, currently predictive models fail to accurately predict necrosis in the retroperitoneum, since almost 30% of patients will harbor teratoma or viable malignancy even with normal post-chemotherapy CT and no teratoma in the primary tumor [9]. A study carried out at the Memorial Sloan-Kettering Cancer Center revealed that 26% of patients had teratoma or viable GCT, even those with radiographically normal retroperitoneum who underwent PC-RPLND [9].

The relatively small number of patients when compared to other institutional retrospective studies is a limitation of the present study; however, other studies with similar sample sizes have also come to significant findings.

In the present study, relative changes in retroperitoneal node size stood out as the single best predictor of the presence of necrosis in the retroperitoneum, which is in accordance with actual logistic regression models [9,10]. While other series suggest 90% as a shrinkage cutoff value to reliably predict necrosis, our quantitative analysis have demonstrated a cutoff level of 35% with approximate sensitivity and specificity [9].

The absence of teratoma has also been demonstrated to be significant in qualitative analysis, but not in quantitative analysis. The presence of choriocarcinoma in the testicular histology predicted necrosis in the retroperitoneum, despite only four (12.5%) patients who had such a finding. This association is an interesting finding that has not been previously reported. We reviewed the literature and no possible explanations were found. We believe further investigation is necessary to confirm this finding, since the number of patients in our sample is relatively small.

## Conclusions

Even though RPLND remains the gold standard for patients with residual masses, those without teratoma in the primary tumor and a shrinkage of 35% or more in retroperitoneal mass have a considerably smaller chance of having viable GCTs or teratoma in the retroperitoneum.

## Abbreviations

AFP: Alphs-fetoprotein; AUC: Area under the curve; CT: Computed tomography; GCT: Germ cell tumor; hCG: Human chorionic gonadotropin; LDH: Lactate dehydrogenase; LVI: Lymph vascular invasion; NSGCT: Nonseminomatous germ cell tumor; PC-RPLND: Post chemotherapy retroperitoneal lymph node dissection; ROC: Receiver operating characteristic; RPLND: Retroperitoneal lymph node dissection.

## Competing interests

The authors declare that they have no competing interests.

## Authors’ contributions

EPM and DKA gathered and compiled data and have been involved in drafting the manuscript. AJN and STL participated in the design of the study and performed the statistical analysis. ACS and MS revised the manuscript critically for important intellectual content. MS and MFD have given final approval of the version to be published. All authors read and approved the final manuscript.
